# Identifying Firearm Violence Exposure in Primary Care Clinical Notes: Protocol for Developing a National Language Processing Text Classifier

**DOI:** 10.2196/76681

**Published:** 2025-09-05

**Authors:** Natalie Carwright, Frances M Biel, Megan Hoopes, Ali Al Bataineh, Pedro Rivera, Kerry Bet, Nicole Cook

**Affiliations:** 1 Department of Mathematics Norwich University Northfield, VT United States; 2 OCHIN Portland, OR United States; 3 David Crawford School of Engineering Norwich University Northfield, VT United States

**Keywords:** firearm violence, firearm injury, natural language processing, electronic health records, text classifier

## Abstract

**Background:**

Structured data codes capture acute bodily injury from firearm violence but do not necessarily describe follow-up care from bodily injury and secondary exposure to firearm violence (eg, witnessing a shooting, being threatened by a firearm, or losing a loved one to gun violence and injury from firearms) even though such exposure is associated with many short- and long-term health impacts. Clinical notes from electronic health records (EHRs) often contain data not otherwise captured in structured data fields and can be categorized using natural language processing (NLP).

**Objective:**

This study protocol outlines the steps being taken to develop an NLP text classifier for determination of exposure to firearm violence (both primary and secondary exposure) from ambulatory primary care and behavioral health EHR clinical notes for persons aged ≥5 years.

**Methods:**

The study will use unstructured data from clinical notes taken between 2012 and 2022 from OCHIN, a multistate network of community health organizations using a single instance of Epic EHR. We describe the process of developing a labeled dataset for supervised NLP development that includes establishing a lexicon (words related to firearm violence) to identify potentially relevant notes, followed by a review of text extracted from a sample of these notes. We then describe the process of building, training, and evaluating candidate machine learning, neural network, and large language model NLP text classifiers. From this, a final NLP model is chosen then evaluated on a new set of randomly selected notes. An engaged stakeholder advisory committee will provide input and guidance on methods and results to identify and address potential biases in the NLP text classifiers.

**Results:**

The study was funded in September 2023. Study activities have been ongoing through July 2025 and we are currently evaluating NLP text classifiers. We expect that the final model will be selected by August 2025 and we will publish results of NLP model development and the final model performance in 2026.

**Conclusions:**

This work describes the development of a novel NLP text classifier to identify exposure to firearm violence in ambulatory primary care and behavioral health clinical notes. The NLP model developed in this study may lead to increased ascertainment of patients with exposure, laying the groundwork for understanding the long-term impacts and outcomes of firearm violence exposure and presenting opportunities for improved patient care.

**International Registered Report Identifier (IRRID):**

DERR1-10.2196/76681

## Introduction

### Background

As injury and death by firearm continues to increase in the United States, so too does secondary exposure to firearm violence (witnessing a shooting, being threatened by a firearm, or the injury or death of a loved one or acquaintance by gun violence). It is estimated that 3 million US children are exposed to firearm violence each year and that 54% of all US adults have experienced firearm violence [1,2]. Primary and secondary exposure can lead to adverse social, emotional, physical, and behavioral health impacts [1,3-6]. Despite the high prevalence of exposure to firearm violence, structured data (eg, diagnostic codes) on firearm violence is largely limited to acute primary exposure (ie, direct injury or death) and there are no International Classification of Diseases (ICD) codes that specifically capture follow-up care from injury or exposure without injury. As a result, most firearm violence research is conducted using acute injury data from hospitals, emergency departments, or death data and is therefore limited to individuals who sustained acute physical injury [7].

Comprehensive data from ambulatory care settings (primary and behavioral health care) are needed to better understand the short- and long-term impacts associated with firearm violence exposure [7-9]. ICD-10 diagnosis codes now exist to document adverse socioeconomic and community circumstances, but these codes are used infrequently across health systems and there is no specific code documenting secondary exposure to gun violence [10,11]. Another opportunity to identify this exposure in clinical settings is through the development and use of custom data collection instruments, such as a trauma history “SmartForm” described in prior work [12]. While these documentation practices show promise, they remain inconsistently available and underused.

Another promising emerging source of data is electronic health record (EHR) clinical notes [13]. Clinical notes include relevant medical care notes that are needed for comprehensive, longitudinal patient health care. Accessing unstructured clinical data using advanced natural language processing (NLP) methods has demonstrated utility in similar types of firearm and trauma research, yet the application of these methods to outpatient settings remains largely unexplored [13-17]. Early work using a keyword search of firearm violence terms identified that exposure to firearm violence is available in unstructured data in ambulatory care settings [18]. This finding provided rationale for developing an NLP text classifier to identify cases of firearm violence exposure documented in clinical notes.

This protocol describes the development of an NLP text classifier to identify firearm violence exposure in primary care and behavioral health care clinical notes. Ambulatory clinics providing primary and behavioral health care offer a novel source of data on firearm violence, particularly exposures that may not have resulted in acute emergency care, hospital encounters, or death. The rich contextual information (eg, patient social history and comorbidities) contained in primary care and behavioral health clinical notes presents an opportunity to expand our understanding of the short- and long-term adverse effects of firearm violence exposure [19-21]. The addition of data from this novel source, combined with learnings from more established data sources on firearm injury and death, could greatly expand our understanding of risks and outcomes associated with exposure [7,22].

### Objective

This research protocol describes the development of a stakeholder-informed NLP model to identify patients with exposure to firearm violence using clinical notes from a large, multistate network of ambulatory health care centers by classifying text as indicative as exposure to firearm violence or not.

## Methods

### Study Aims and Design

This protocol describes the development of a stakeholder-informed NLP text classifier to identify patients with firearm violence exposure documented in unstructured clinical notes during a primary care or behavioral health care encounter. The current study builds on pilot work that confirmed the presence of unstructured data in clinical notes that included firearm violence keywords that had contextual meaning of exposure [18]. Study findings, when available, will be reported following guidance for reporting machine learning model development in biomedical research [23].

### Setting

The data used in this study come from OCHIN, a multistate network of community health organizations using a single instance of Epic EHR [24]. OCHIN is a nonprofit health care innovation center that offers a fully hosted, highly customized instance of Epic practice management and EHR solutions at over 2000 health care delivery sites across 40 states. OCHIN member health organizations provide health care services to patients in low-resource settings, regardless of their ability to pay. We will access clinical notes from unstructured records in natural language serving as a record of treatment, diagnoses, and clinical progress from ambulatory primary care and behavioral health care encounters to patients aged ≥5 years at encounter from January 1, 2012, to December 31, 2022. These chart notes will be used to develop an NLP model that will classify the note text as representative of firearm violence exposure or not.

### Ethics Approval

OCHIN community health organizations, as part of their contract with OCHIN, authorize OCHIN to create limited datasets of member information for certain research activities consistent with applicable law and aligning with best practices of research, including institutional review board oversight of study activities.

This study was reviewed and deemed exempt by the Norwich University Institutional Review Board. Only research staff who are OCHIN employees have access to clinical notes, and efforts were made to remove direct patient identifiers prior to review.

### Data Source

Data for NLP model development will be identified by searching for keyword phrases in clinical notes for study-eligible patient encounters. We define study-eligible patient encounters as those occurring between January 1, 2012, and December 31, 2022, with patients who were aged ≥5 years on their date of encounter (4,024,926 patients). This study period aligns with the date range of EHR data availability at the time of study start. The age restriction was imposed to reduce the number of “false positive” firearm exposure measures resulting from screening for household gun ownership commonly assessed in early childhood encounters [25]. We further restricted eligible ambulatory encounters to those categorized as either primary or behavioral health, using a previously defined algorithm (31,801,530 encounters) [26].

### Stakeholder Advisory Committee

The NLP model development will be guided by an 8- to 10-person stakeholder advisory committee (SAC), a multidisciplinary group of individuals who will review methods, provide guidance on testing and evaluation, participate in interpreting and disseminating study findings, and evaluate the social and ethical merits for ongoing study and model deployment. SAC members include people with lived experience of firearm violence exposure, clinicians, researchers, clinical informaticists, and data scientists.

Development of the SAC will be conducted during the first 3 months of the study. SAC members will meet monthly, alternating between synchronous and asynchronous meeting formats, to guide study activities and will be compensated for their time (the mean compensation will be $185 per hour). Due to the dynamic nature of the research activities and the novel application of a SAC for NLP development around firearm violence, the ongoing contribution of SAC members after lexicon development and review (see below) will continue to evolve as study activities progress. We anticipate that SAC members will participate in interpreting research findings and provide ongoing input into the applicability of the model in clinical practice.

### Dataset Development

During pilot work, a list of keywords informed by MacPhaul et al [13] was applied to a set of notes for patients with known exposure to firearm violence. A random sample of 30 notes from each of 3 text string search categories (broad, gun-only, and shooting) were reviewed to contextualize how keywords are used in clinical notes and to identify lexicon refinements (eg, remove preset template EHR phrases and add keywords[18]). Among the 90 notes reviewed, 13 (14%) were determined to be true “cases,” while all other notes did not indicate exposure to or injury from firearm violence.

In this study, the lexicon (Multimedia Appendix 1) was expanded to include additional terms used by Chew et al [27] and will be used to extract a random sample of 5000 clinical notes that contain keywords for review. Applying a case rate of 14%, we anticipate that 700 of these clinical notes will indicate exposure to firearm violence. The study team worked together to develop a case note selection decision guide to clarify inclusion and exclusion criteria and reduce variation among study team annotators (Multimedia Appendix 2). The guide defines a case as “primary or secondary exposure to firearm violence as a direct witness of firearm violence or the acute aftermath.” The decision guide clarifies that cases will include any mention in the clinical note of gun threats (primary or secondary) experienced by a patient. In addition, cases include mention of retained bullets in the body, sequalae from firearm violence, patients brandishing or perpetrating crime with a gun, or self-inflicted gunshot wounds. Following pilot methods, the sample of clinical notes will be posted as a shared Microsoft Excel document for review. Study team members independently review notes for keywords and contextualize the meaning of the note as case or noncase, copying over relevant sentences that informed their decision into the document. When case status is not apparent from the clinical note even though it contains lexicon words, the reviewer will indicate in an electronic comment that additional review and adjudication is needed. An additional 2 study team reviewers will review the clinical note, document their assessment, and the 3 reviewers will subsequently meet to make a determination. If additional input is needed for categorization the study team will involve the clinician informaticists on the study team, one of whom is a primary care physician and the other a behavioral health clinician. If needed, the decision guide will be updated iteratively by the study team. Counts of total notes available, the prevalence of keywords, and the prevalence of firearm violence–indicative notes will be recorded and detailed in future publications.

The study team will also engage the SAC to review keywords and suggest additional terms. These terms will be evaluated for inclusion into the lexicon by performing a keyword search to identify potentially related notes, followed by a review of a sample (up to 30 per term, if available in notes) of these notes. Terms that identify at least one note indicative of firearm violence exposure will be added to the lexicon. Engaging stakeholders is an important approach for incorporating broad community perspectives in developing and applying NLP and other artificial intelligence in health care, reducing bias and improving applicability [28-30].

### NLP Text Classifier Development

The adjudicated notes will become the labeled dataset for training and testing our NLP text classifier. The study team will use Python (Python Software Foundation) to build the NLP text classifier. The output of our classifier will result in a new structured data variable that identifies exposure to firearm violence from behavioral health or primary care encounters in ambulatory settings.

The NLP text classifier will be trained as follows:

1. The dataset, containing chart notes and the adjudicated label, will be randomly separated into training and test sets using an 80% to 20% train-test split, stratified by label.
2. The study team will use the training set to train several baseline machine learning (ML) and neural network (NN) classifiers available in the *scikit-learn* Python library [31] and Tensorflow platform [32] with Keras application programming interface [33], respectively, implemented with their default values.
  Baseline ML models will consist of a pipeline with a vectorizer and classifier. The vectorizer will be a term frequency inverse document frequency [34] implemented via *scikit-learn’s* TfidfVectorizer. ML classifiers will include random forest, multinomial naïve Bayes, gradient boosting, and LightGBM, implemented using *scikit-learn* and the LightGBM library [35].
  Baseline NN models will use a tokenizer with a 1000-word vocabulary fit, and transformed sequences will be padded to 95% of the maximum sequence length. A sequential model consisting of an embedding layer with dimension 100, a model layer, and a dense layer with sigmoid activation will be compiled using an Adam optimizer, binary cross entropy loss, 50 epochs, a batch size of 64, and a validation split of 0.20. Model layers will be either recurrent neural network, long short-term memory, or gated recurrent unit with 64 units.
3. Performance of baseline models will be measured by evaluation on the held-out test set using metrics such as precision, recall, specificity, and the *F*_1_-score (in future work, we would instead use cross-validation on the training set to assess baseline model performance to avoid data leakage).
4. The ML and NN baseline models with the higher evaluation metrics on the held-out test set will then be tuned for model hyperparameters, architecture (NN), learning parameters (NN), and threshold.
  Tuning of the ML model will use BayesSearchCV from the *scikit-optimize* library [31] using the vectorizer-classifier pipeline with cross validation and 60 iterations and scored using average precision. The model with the best parameters will be fit on the training and validation data and then the optimal threshold will be determined using sckikit-learn’s TunedThresholdClassifierCV with cross validation and the *F*_1_-score.
  Tuning of the NN model will use a text vectorization layer with tunable max_tokens and output_sequence_length; an embedding layer with tunable output_dim; a tunable number of NN layers each followed by a dropout layer with a tunable number of units, activation function, and dropout rate; and lastly, a dense layer with sigmoid activation. The model will be compiled using an Adam optimizer and binary cross entropy loss. The model will be fit with a tunable batch size. The training set will be split into training and validation sets and the BayesianOptimization tuner will be used to tune hyperparameters using 60 trials with 3 executions per trial, an objective of maximizing the average precision score, 100 epochs, and early stopping with patience=2. The model with the best parameters will again be fit on all the training data for 100 epochs, and the epoch with the maximum average precision score on the validation split will be assigned as the optimal number of epochs. The model with the best parameters will be fit on the training and validation data using this optimal number of epochs. The optimal threshold will be determined using cross validation and the *F*_1_-score.
5. An open-source large language model, clinicalBERT [36], will be fine-tuned using the training and validation sets and the Transformers [37] and Datasets [38] libraries from HuggingFace to produce another NLP text classifier. The learning rate, batch size, and number of epochs will be tuned manually, using initial values of 0.00005, 16, and 5, respectively.
6. Evaluation of the tuned models and the clinicalBERT model will be done by assessing evaluation metrics of model predictions on the held-out test set; assessing evaluation metrics of model predictions on the held-out test set across subpopulations defined by age, sex, and race or ethnicity, and the intersection of these subpopulations; reviewing and discussing the performance of tuned and clinicalBERT models with the SAC.
7. Documentation of model performance and suggestions for any preprocessing, inprocessing, or postprocessing methods that should be pursued to mitigate any observed biases in the predictions of tuned or clinicalBERT models on the test set will be carried out.
8. One of the tuned models or the clinicalBERT model will be selected as our final NLP model, using the criteria in step 6.
9. Evidence of the validity of the final NLP model will be collected. We will do this by applying a keyword search to extract text excerpts from 1,000,000 randomly selected novel notes not previously adjudicated or used. From preliminary data pulls, we estimate that approximately 3% (n=30,000) of notes will have a keyword. Our final NLP model will be applied to these text excerpts, and a random sample of these (n=1000) will be adjudicated to give evidence as to the validity of the model. Adjudication will follow the same process described in the Dataset Development section above.

### Addressing Ethics, Equity, and Bias in Model Development

The study team is approaching development of the NLP text classifier with a lens toward minimizing biases that may emerge [39-41]. Our primary strategy to address bias in model development is through the engagement of a SAC throughout the study. SAC members will participate in discussions about the use of an NLP-based classifier in a clinical context. Discussions with SAC members on the implications of false positives and false negatives at both the patient level and research findings level will help guide how potential NLP models will be evaluated (ie, which metrics are of higher importance). The SAC will be asked to consider bias based on patient characteristics and provider practices as well as algorithmic bias. Inclusion of SAC-suggested terms indicative of firearm violence and considerations of model performance across subpopulations are key evaluative characteristics for considering, identifying, and then mitigating potential biases in a universal firearm violence NLP algorithm. For additional diversity in input, our research team will meet with OCHIN’s primary care and behavioral health clinical workgroups, attended by clinical leaders from member health organizations during the study start up (Year 1, quarter 1).

Potential biases in our training data include missing patients where firearm violence exposure is not present in a clinical note, possibly due to care received outside the health center, variation in provider care, or documentation practices, and patients who do not self-report exposure. These biases are inherent to all EHR-based research, and generalizability should be interpreted in light of the convenience-based, albeit large, nature of the dataset used [42,43] Our data come from primarily low-resource populations but it is not necessarily representative of all subgroups or individuals most likely to experience exposure to firearm violence. Likewise, we do not have reliable data on many variables in which model bias may exist (eg, gender).

There is the possibility of annotation bias during adjudication of text excerpts from clinical notes, even with our experienced team members. To minimize this bias, the study team developed a case note selection decision guide to support review by different study team members. We did not assess inter-rater reliability but would recommend this be performed in future projects.

We will attempt to identify potential model bias by calculating model performance across subpopulations of sex, age, race and ethnicity, and the intersections of these subpopulations (ie, group fairness) as these are protected attributes that are reliably documented in our data. We will provide this information to the SAC for further consideration along with discussions of stereotypical bias that can occur by evaluating models over such groups, under-representation bias due to low numbers in some subgroups, and possible mitigation strategies.

There may be other types of biases present in an NLP model that require more in-depth analysis to identify and mitigate. Such analyses include counterfactual evaluation (eg, replacing pronouns), adversarial testing, checking for bias in word embeddings, and evaluating model bias on an external dataset. However, due to the short duration of this study that spans lexicon development to the evaluation of NLP models, and without the intent to put this model in production at the end of this study, we leave these advanced and necessary bias identification methods to future work. If we do identify any biases in this study, we will document and provide transparency to these biases. We do not foresee time to effectively mitigate all potential biases within this study period. If there are identified biases and time left in our study period, we will consider and use appropriate mitigation strategies, which will most likely involve postprocessing mitigation techniques such as calibration and threshold adjustment given time constraints.

## Results

The AIM-AHEAD Impact of Gun Violence Exposure on Health was funded on October 1, 2023, for a 2-year period.

### NLP Model Development

Data acquisition started in February 2024 and was completed in April 2024, with >85 million primary care and behavioral health notes from >7 million patients. The study team extracted text from a random sample of 5000 notes with at least 1 firearm violence term for review and adjudication between March 2024 and May 2024. The first NLP template for baseline models was run in July 2024. Model development will continue throughout the study period to identify the best performing model for potential deployment. We expect that the final model will be selected by August 2025, and we will publish results of NLP model development and the final model performance in 2026.

### Stakeholder Advisory Committee

The SAC was established during the first 3 months of the study in 2023 and includes 12 members: 5 community advocates or patients with lived experience; 1 physician; 2 clinician researchers; 2 clinical informaticists; and 2 data scientists with firearm violence data and research experience. The SAC will continue to meet monthly for the duration of the study. SAC contribution includes providing guidance on lexicon development, review of NLP performance, and application of NLP model in clinical practice [30].

## Discussion

While exposure to firearm violence is common, identification of this experience is rare in structured ambulatory EHR data, particularly for secondary exposure that does not result in acute bodily injury. This study will be the first to develop an NLP text classifier to identify firearm violence exposure in clinical notes. We expect the NLP model developed in this study will be able to increase ascertainment of primary care and behavioral health patients with exposure to firearm violence; these findings will lay the groundwork for understanding the long-term impacts and outcomes of firearm violence exposure and present opportunities for improved patient care. Engagement with OCHIN network providers throughout the study will facilitate integration of results with clinical care and documentation practices. NLP has been used in other contexts to identify processes poorly captured by ICD codes; we expect that our work will also be able to identify patterns of exposure not otherwise captured in patient EHRs [44-46].

Because of the novelty of applying NLP to clinical notes for this purpose, we intentionally kept our approach open-ended and did not define a precise taxonomy or operational definition for firearm violence exposure, nor will we attempt to delineate between different types of exposure (eg, witnessing a community event versus direct personal threat). This is a limitation and presents important opportunities for future study and refinement. On the other hand, clinical notes are unlikely to represent a comprehensive picture of firearm violence exposure, and documentation is likely biased by provider practices and patient characteristics [43]. The value of identifying patients with exposure to firearm violence is to alert providers to the potential health care and support needs of such patients, to expand our knowledge of health sequalae following firearm violence, and to more fully frame the public health burden of firearm violence. Future work would benefit from collating clinical note–based NLP models with other sources of firearm violence exposure information—such as ICD-10 codes, personal and family history, and trauma screening—documented within and across clinical settings.

Additional limitations include the exclusion of clinical notes from children younger than 5 years, which limits generalizability, and the lack of more recent data, which may yield differential documentation patterns in structured or unstructured data. These limitations may be addressed in future studies.

Ultimately, addressing the epidemic of gun violence in the US and understanding the long-term impacts of exposure will require more comprehensive data. The NLP model developed through this work represents one aspect of this unmet need [7-10].

## Appendix

Multimedia Appendix 1 Firearm keywords.

Multimedia Appendix 2 Case note selection decision guide.

## Abbreviations

ADVANCE: Accelerating Data Value Across a National Community Health Center
EHR: electronic health record
ICD: International Classification of Diseases
ML: machine learning
NLP: natural language processing
NN: neural network
SAC: stakeholder advisory committee

## Data Availability

Because chart notes contain protected health information, raw data for this study are not accessible to researchers outside of OCHIN. Model natural language processing code is available upon request.

## References

[1] Finkelhor D, Turner HA, Shattuck A, Hamby SL (2015). Prevalence of childhood exposure to violence, crime, and abuse: results from the National Survey of Children's Exposure to Violence. JAMA Pediatr.

[2] Schumacher S, Kirzinger A, Presiado M, Valdes I, Brodie M (2023). Americans’ experiences with gun-related violence, injuries, and deaths. KFF.

[3] Mitchell KJ, Jones LM, Turner HA, Beseler CL, Hamby S, Wade R (2021). Understanding the impact of seeing gun violence and hearing gunshots in public places: findings from the Youth Firearm Risk and Safety Study. J Interpers Violence.

[4] Turner HA, Mitchell KJ, Jones LM, Hamby S, Wade R, Beseler CL (2019). Gun violence exposure and posttraumatic symptoms among children and youth. J Trauma Stress.

[5] Smith ME, Sharpe TL, Richardson J, Pahwa R, Smith D, DeVylder J (2020). The impact of exposure to gun violence fatality on mental health outcomes in four urban U.S. settings. Soc Sci Med.

[6] Ranney M, Karb R, Ehrlich P, Bromwich K, Cunningham R, Beidas RS, FACTS Consortium (2019). What are the long-term consequences of youth exposure to firearm injury, and how do we prevent them? A scoping review. J Behav Med.

[7] Cook N, Sills M (2023). Tracking all injuries from firearms in the US. JAMA.

[8] Kaufman EJ, Delgado MK (2022). The epidemiology of firearm injuries in the US: the need for comprehensive, real-time, actionable data. JAMA.

[9] Kaufman EJ, Delgado MK (2023). Tracking all injuries from firearms in the US-reply. JAMA.

[10] Llamocca EN, Ahmedani BK, Lockhart E, Beck AL, Lynch FL, Negriff SL, Rossom RC, Sanchez K, Sterling SA, Stults C, Waring SC, Harry ML, Yu H, Madziwa LT, Simon GE (2025). Use of codes for adverse social determinants of health across health systems. Psychiatr Serv.

[11] ICD-coding of firearm injuries. National Center for Health Statistics.

[12] Cook N, Hoopes M, Biel FM, Cartwright N, Gordon M, Sills M (2024). Early results of an initiative to assess exposure to firearm violence in ambulatory care: descriptive analysis of electronic health record data. JMIR Public Health Surveill.

[13] MacPhaul E, Zhou L, Mooney SJ, Azrael D, Bowen A, Rowhani-Rahbar A, Yenduri R, Barber C, Goralnick E, Miller M (2023). Classifying firearm injury intent in electronic hospital records using natural language processing. JAMA Netw Open.

[14] Goldstein EV, Mooney SJ, Takagi-Stewart J, Agnew BF, Morgan ER, Haviland MJ, Zhou W, Prater LC (2023). Characterizing female firearm suicide circumstances: a natural language processing and machine learning approach. Am J Prev Med.

[15] Zafari H, Kosowan L, Zulkernine F, Signer A (2021). Diagnosing post-traumatic stress disorder using electronic medical record data. Health Informatics J.

[16] Brandt C, Workman TE, Farmer M, Akgün KM, Abel E, Skanderson M, Bean-Mayberry B, Zeng-Treitler Q, Mason M, Bastian L, Goulet J, Post L (2021). Documentation of screening for firearm access by healthcare providers in the veterans healthcare system: a retrospective study. West J Emerg Med.

[17] Trujeque J, Dudley R, Mesfin N, Ingraham NE, Ortiz I, Bangerter A, Chakraborty A, Schutte D, Yeung J, Liu Y, Woodward-Abel A, Bromley E, Zhang R, Brenner LA, Simonetti JA (2025). Comparison of six natural language processing approaches to assessing firearm access in Veterans Health Administration electronic health records. J Am Med Inform Assoc.

[18] Cook N, Biel F, Cartwright N, Hoopes M, Al Bataineh Ali, Rivera P (2024). Assessing the use of unstructured electronic health record data to identify exposure to firearm violence. JAMIA Open.

[19] DeVoe JE, Gold R, Cottrell E, Bauer V, Brickman A, Puro J, Nelson C, Mayer KH, Sears A, Burdick T, Merrell J, Matthews P, Fields S (2014). The ADVANCE network: accelerating data value across a national community health center network. J Am Med Inform Assoc.

[20] Gold R, Kaufmann J, Cottrell E, Bunce A, Sheppler CR, Hoopes M, Krancari M, Gottlieb LM, Bowen M, Bava J, Mossman N, Yosuf N, Marino M (2023). Implementation support for a social risk Screening and referral process in community health centers. NEJM Catal Innov Care Deliv.

[21] Gruß I, Bunce A, Davis J, Dambrun K, Cottrell E, Gold R (2021). Initiating and implementing social determinants of health data collection in community health centers. Popul Health Manag.

[22] Fontanarosa PB, Bibbins-Domingo K (2022). The unrelenting epidemic of firearm violence. JAMA.

[23] Luo W, Phung D, Tran T, Gupta S, Rana S, Karmakar C, Shilton A, Yearwood J, Dimitrova N, Ho TB, Venkatesh S, Berk M (2016). Guidelines for developing and reporting machine learning predictive models in biomedical research: a multidisciplinary view. J Med Internet Res.

[24] Ochin.

[25] Dowd M, Sege R, Council on Injury‚ Violence‚Poison Prevention Executive Committee, American Academy of Pediatrics (2012). Firearm-related injuries affecting the pediatric population. Pediatrics.

[26] Cook N, McGrath BM, Navale SM, Koroukian SM, Templeton AR, Crocker LC, Zyzanski SJ, Bensken WP, Stange KC (2024). Care delivery in community health centers before, during, and after the COVID-19 pandemic (2019-2022). J Am Board Fam Med.

[27] Chew RF, Weitzel KJ, Baumgartner P, Oppenheimer WC, Liu S, Miller AB, Lowe A, Yaros AC, D'Arcangelo B, Barnes A Improving text classification with Boolean retrieval for rare categories: a case study identifying firearm violence conversations in the Crisis Text Line database. RTI Press.

[28] Vishwanatha JK, Christian A, Sambamoorthi U, Thompson EL, Stinson K, Syed TA (2023). Community perspectives on AI/ML and health equity: AIM-AHEAD nationwide stakeholder listening sessions. PLOS Digit Health.

[29] Wan R, Kim J, Kang D (2023). Everyone’s voice matters: quantifying annotation disagreement using demographic information. Proc AAAI Conf Artif.

[30] Cook N, Biel FM, Bet KA, Sills MR, Al Bataineh A, Rivera P, Templeton AR, Cartwright N (2025). Engaging stakeholders with professional or lived experience to improve firearm violence lexicon development. JMIR Form Res.

[31] Pedregosa F, Varoquaux G, Gramfort A, Michel V, Thirion B, Grisel O, Blondel M, Prettenhofer P, Weiss R, Dubourg V, Vanderplas J, Passos A, Cournapeau D, Brucher M, Perrot M, Duchesnay É (2011). Scikit-learn: machine learning in Python. Journal of Machine Learning Research.

[32] Abadi M, Agarwal A, Barham P, Brevdo E, Chen Z, Citro C, Corrado GS, Davis A, Dean J, Devin M, Ghemawat S, Goodfellow I, Harp A, Irving G, Isard M, Jia Y, Jozefowicz R, Kaiser L, Kudlur M, Levenberg J, Mane D, Monga R, Moore S, Murray D, Olah C, Schuster M, Shlens J, Steiner B, Sutskever I, Talwar K, Tucker P, Vanhoucke V, Vasudevan V, Viegas F, Vinyals O, Warden P, Wattenberg M, Wicke M, Yu Y, Zheng X TensorFlow: large-scale machine learning on heterogeneous distributed systems. ArXiv.

[33] (2015). Keras. GitHub.

[34] Sparck Jones K (1972). A statistical interpretation of term specificity and its application in retrieval. J Doc.

[35] LightGBM R-package. lightgmb.

[36] Huang K, Altosaar J, Ranganath R Clinicalbert: modeling clinical notes and predicting hospital readmission. ArXiv.

[37] Wolf T, Debut L, Sanh V, Chaumond J, Delangue C, Moi A, Cistac P, Rault T, Louf R, Funtowicz M, Davison J, Shleifer S, von Platen P, Ma C, Jernite Y, Plu J, Xu C, Le Scao T, Gugger S, Drame M, Lhoest Q, Rush A (2020). Transformers: state-of-the-art natural language processing.

[38] Lhoest Q, Villanova del Moral A, Jernite Y, Thakur A, van Platen P, Patil S, Chaumond J, Drame M, Plu J, Tunstall L, Davison J, Šaško M, Chhablani G, Malik B, Brandeis S, Le Scao T, Sanh V, Xu C, Patry N, McMillan-Major A, Schmid P, Gugger S, Delangue C, Matussière T, Debut L, Bekman S, Cistac P, Goehringer T, Mustar V, Lagunas F, Rush A, Wolf T (2021). Datasets: a community library for natural language processing.

[39] Char DS, Shah NH, Magnus D (2018). Implementing machine learning in health care - addressing ethical challenges. N Engl J Med.

[40] Chen Y, Clayton EW, Novak LL, Anders S, Malin B (2023). Human-centered design to address biases in artificial intelligence. J Med Internet Res.

[41] Wiens J, Saria S, Sendak M, Ghassemi M, Liu VX, Doshi-Velez F, Jung K, Heller K, Kale D, Saeed M, Ossorio PN, Thadaney-Israni S, Goldenberg A (2019). Do no harm: a roadmap for responsible machine learning for health care. Nat Med.

[42] Beach MC, Saha S, Park J, Taylor J, Drew P, Plank E, Cooper LA, Chee B (2021). Testimonial injustice: linguistic bias in the medical records of Black patients and women. J Gen Intern Med.

[43] Perets O, Stagno E, Yehuda E, McNichol M, Anthony Celi L, Rappoport N, Dorotic M Inherent bias in electronic health records: a scoping review of sources of bias. MedRXiv.

[44] Sheikhalishahi S, Miotto R, Dudley JT, Lavelli A, Rinaldi F, Osmani V (2019). Natural language processing of clinical notes on chronic diseases: systematic review. JMIR Med Inform.

[45] Ridgway JP, Uvin A, Schmitt J, Oliwa T, Almirol E, Devlin S, Schneider J (2021). Natural language processing of clinical notes to identify mental illness and substance use among people living with HIV: retrospective cohort study. JMIR Med Inform.

[46] Chae S, Song J, Ojo M, Topaz M (2021). Identifying heart failure symptoms and poor self-management in home healthcare: a natural language processing study. Stud Health Technol Inform.

