# Supplementary material for: Identifying Firearm Violence Exposure in Primary Care Clinical Notes: Protocol for Developing a National Language Processing Text Classifier
Source: JMIR Res Protoc. 2025 Sep 5;14:e76681. doi: 10.2196/76681 (PMC12449666; doi:10.2196/76681)
Supplement: Multimedia Appendix 1 [file resprot_v14i1e76681_app1.docx]

Multimedia Appendix 1: [firearm keywords]

Included terms and Phrases in 5000 data pull

gun, firearm, pistol, handgun, weapon, shot, shotgun, rifle, glock, revolver, shooter, bullet, GSW, gunshot, shooting, packing heat
