# Supplementary material for: Identifying Firearm Violence Exposure in Primary Care Clinical Notes: Protocol for Developing a National Language Processing Text Classifier
Source: JMIR Res Protoc. 2025 Sep 5;14:e76681. doi: 10.2196/76681 (PMC12449666; doi:10.2196/76681)
Supplement: Multimedia Appendix 2 [file resprot_v14i1e76681_app2.docx]

Multimedia Appendix 2: [case note selection decision guide]

NLP Case note selection decision guide

Definition: primary or secondary exposure to firearm violence (direct witness of firearm violence or the acute aftermath)

- Includes:
  - Any instance (mention) of gun threats (primary or secondary) experienced by a patient
    - Secondary exposure indicates family member, friend, in their community, etc.
  - Retained bullet
  - Sequelae from gunshot wounds
  - Patient self-inflicted gunshot wound or patient acting as perpetrator for GV instance
    - Includes patient brandishing gun as threat to others
    - Includes patient using a firearm as part of a robbery or DV incident as perpetrator
  - Patient indication of suicidal attempt with gun
- Excludes:
  - Access to, or ownership of, firearms where there is no mention of primary or secondary threat due to firearm or exposure to firearm violence.
  - Individuals who indicate GV exposure within the context of delusions
  - Patient making verbal threats of GV to others without indication of wielding gun during said threat
  - Mention of bullet journaling or bulletproof coffee
  - Weapons charges without context to type of weapon
    - E.g. ‘Assault with a deadly weapon’ is to be excluded
    - E.g. ‘hit on head with a deadly weapon’ is to be excluded

Instructions for isolating sections of note text

- Isolate the section of text that indicates the GV exposure status.
  - If it is a form element (e.g. ‘Access to firearms’), then only copy the relevant form element and not surrounding text. Do not copy the YES/NO responses, since we have determined above that access to firearms is NOT an indicator of GV exposure.
  - If the text is part of a single sentence, copy the single sentence.
  - If text exists outside of one sentence that would give context to the status of the GV exposure, then copy the relevant section of text (1 – 3 sentences).
- If more than one section of text in an individual note indicates exposure, copy those sections over following the above guidelines.
  - Separate these sections with a carriage repeat. In Excel, select ALT + ENTER to move to a new line in a cell.
- Do not copy over repeated instances of the same text, if the note itself is duplicated within an individual cell.
